# Review of the tribe Chilocorini Mulsant from Iran (Coleoptera, Coccinellidae)

**DOI:** 10.3897/zookeys.712.20419

**Published:** 2017-10-31

**Authors:** Amir Biranvand, Wioletta Tomaszewska, Wenjing Li, Vincent Nicolas, Jahanshir Shakarami, Lida Fekrat, Shahram Hesami

**Affiliations:** 1 Young Researchers and Elite Club, Khorramabad Branch, Islamic Azad University, Khorramabad, Iran; 2 Museum and Institute of Zoology, Polish Academy of Sciences, Warszawa, Poland; 3 Plant Protection Research Institute, Guangdong Academy of Agricultural Sciences, Guangzhou, Guangdong 510640, China; 4 27 Glane, 87200 Saint-Junien, France; 5 Plant Protection Department, Lorestan University, Agricultural faculty, Khorramabad, Iran; 6 Department of Plant Protection, Faculty of Agriculture, Ferdowsi University of Mashhad, Mashhad, Iran; 7 Department of Entomology, College of Agricultural Sciences, Shiraz Branch, Islamic Azad University, Shiraz, Iran

**Keywords:** checklist, Chilocorini, Coccinelloidea, Iran, review

## Abstract

The Iranian checklist of the tribe Chilocorini Mulsant, 1846 (Coleoptera: Coccinellidae) is updated. In total, 13 species belonging to four genera (*Brumoides* Chapin, 1965, *Chilocorus* Leach, 1815, *Exochomus* Redtenbacher, 1843, and *Parexochomus* Barovsky, 1922) are listed from Iran. An identification key to all genera and species currently known from Iran is presented along with illustrations of adult specimens and male genitalia.

## Introduction

The family Coccinellidae, with nearly 6000 species and 360 genera, belongs currently to the superfamily Coccinelloidea (Coleoptera: Polyphaga) (Robertson et al. 2015, Tomaszewska and Szawaryn 2016). It is divided into two subfamilies: Microweiseinae and Coccinellinae. The subfamily Microweiseinae includes three tribes: Carinodulini, Microweiseini (including Sukunahikonini) and Serangiini (Escalona and Ślipiński 2012); the remaining taxa belong to the subfamily Coccinellinae (Seago et al. 2011, Robertson et al. 2015, Szawaryn et al. 2015, Escalona et al. 2017).

The tribe Chilocorini Mulsant, 1846 contains approximately 250 species belonging to 27 genera (Łączyński and Tomaszewska 2012, Li et al. 2017), of which nine genera have hitherto been recorded from Palaearctic region including: *Brumoides* Chapin, 1965, *Chilocorus* Leach, 1815, *Chujochilus* Sasaji, 2005, *Exochomus* Redtenbacher, 1843, *Parexochomus* Barovsky, 1922, *Phaenochilus* Weise, 1895, *Priscibrumus* Kovár, 1995, *Simmondsius* Ahmad & Ghani, 1966 and *Xanthocorus* Miyatake, 1970) (Kovář 2007).

Although most members of Chilocorini are coccidophagous (Giorgi et al. 2009, Escalona et al. 2017), aphidophagy is also present in some species (Ślipiński and Giorgi 2006); so, the members of this tribe have the potential to be effective biological control agents of coccids and aphids (Drea and Gordon 1990, Ponsonby and Copland 1997).

In the last classification of the former subfamily Chilocorinae by Kovář (2007), the species of the genus *Brumus* Mulsant, 1850 were transferred to Exochomus Redtenbacher and the subgenus Parexochomus of *Exochomus* was considered as a valid genus, under the name of *Parexochomus* Barovsky, 1922. This classification was followed by Nedvěd and Kovář (2012). Moreover, according to Ślipiński (2007), the subfamily Chilocorinae Mulsant was dissolved and all tribes were lumped into the subfamily Coccinellinae. This classification was confirmed by subsequent morphological and molecular studies (Seago et al. 2011, Robertson et al. 2015). The number of genera and species of this tribe is continuously increasing (Ślipiński and Giorgi 2006, Łączyński and Tomaszewska 2009, Wang and Ren 2010, Łączyński and Tomaszewska 2012, Li et al. 2015, Li et al. 2017) and it is expected that this trend will be continuing.

Although a large number of species of this tribe have hitherto been reported from Iran (Duverger 1983, Kovář 2007, Moddarres-Awal 2012), there is no complete and comprehensive information on the Iranian Chilocorini. The checklist by Abdolahi Mesbah et al. (2016) differs from our view and does not include identification key, diagnosis, and synonymy. Our paper corrects the previous studies on the species of this tribe in Iran, in order to update the information about Iranian Chilocorini.

## Materials and methods

This study was mainly based on review of the literature along with the samples collected by the first author. The samples were collected by hand, aspirator, or sweep net in the fields, orchards, and pastures of various provinces of Iran. The specimens were examined under Olympus stereomicroscope (SZ-ST). The specimens were first boiled in 10% KOH for a maximum of 20 min depending on the darkness of the body color/ sclerotization in order to dissect the genitalia. The dissected genitalia were then transferred into distilled water for a maximum of 10 min to rinse off the KOH. Finally, the slides were prepared using Canada balsam. The slides were examined under a microscope (Olympus CX21) and images were taken using a digital camera and edited in Photoshop software (Adobe Photoshop CS5.1). The specimens were identified to species using available keys and resources (Mader 1955, Fürsch 1961, Bielawski 1984, Kovář 1995, Raimundo and van Harten 2000, Raimundo et al. 2008).

Although the higher classification of Seago et al. (2011) was followed in this study, taxonomy at the species level is based on Kovář (2007). Morphological terminology follows that of Ślipiński (2007). All of the specimens collected and examined during this study are deposited in Plant Protection Department, Lorestan University, Agricultural Faculty, Khorramabad, Iran.

## Results and discussion

The Iranian coccinellid species list of the tribe Chilocorini is updated, which includes 13 species belonging to four genera (*Brumoides*, *Chilocorus*, *Exochomus*, and *Parexochomus*).

Although there are some records of *Exochomus flavipes* Thunberg, 1781 from Iran (Ansari pour and Shakarami 2011, Tavakol et al. 2014), re-examination of the voucher specimens of this species showed that these reports are misidentifications and these samples are actually *Parexochomus nigromaculatus* (Goeze, 1777). *Parexochomus flavipes* is morphologically similar to *P. nigromaculatus* but is distinguished from it by the male genitalia, and *P. flavipes* has not hitherto been reported from Palaearctic region (Kovář 2007). It is distributed in the northern states of USA (Gordon 1985) and south and west of Africa (Fürsch 1961).

Mahghari and Ostovan (2006) reported two ladybird species, *Brumus undecempunctata* L. and *Chilocorus stigma* (Say, 1835), from the northern provinces of Iran (Gilan and Mazandaran province) as natural enemies of whiteflies. In coccinellid taxonomy, there is no known species under the name of *Brumus undecempunctata*, while *Chilocorus stigma* has not been reported so far from Palaearctic region (Kovář 2007). According to our knowledge, the presence of these species in Iran is doubtful and not confirmed.

Barovsky (1922) reported *Exochomus kiritshenkoi* Barovsky, 1922 from Iran (Shahrood, H. Christoph leg.). There are also specimens in Zoologichesky Institut (Akademii Nauk SSSR) in St. Petersburg, labeled as *E. kiritshenkoi* which had been collected from Iran (Shahrood, H. Christoph leg). Kovář (1995) however identified these specimens as *E. gebleri* Weise.

Data on the presence of *E. bifasciatus* in Iran are based on Kovář (2007). Since we do not have any information (particularly morphological) about this species, it is excluded from the identification key of Iranian species of Chilocorini.

### Subfamily Coccinellinae Latreille, 1807

#### Tribe Chilocorini

Taxon classificationAnimaliaColeopteraCoccinellidae

Mulsant, 1846

##### Diagnosis.

Body size small to medium (2.0–8.0 mm), with downward directed head inserted into prothorax to some extent; dorsum usually without obvious pubescence. Head wider than long, flattened ventrally; clypeus variously expanded laterally and wholly concealing antennal insertions. Mandibles triangular, strong with an apical tooth and heavily developed molar teeth; maxillary palps relatively long, terminal palpomere parallel sided to weakly enlarged apically; labial palp clearly separated basally, inserted on ventral side of prementum. Antenna composed of 7–10 antennomeres, markedly short with a fusiform club composed of three terminal antennomeres. Prosternum fairly elongate in front of coxae; prosternal process narrow, parallel sided without carinae. Hind wings with large anal lobe. Elytra irregularly punctate, with epipleuron wide and complete to apex, frequently with foveae for receiving apices of femora. Abdomen with five or six ventrites; postcoxal lines at abdominal ventrite 1 variable, without associated pits and pores. Male genitalia with symmetrical tegmen, penis guide sometimes asymmetrical; parameres well developed, apically setose; penis a simple, single sclerite with sizeable basal capsule. Coxites triangular and faintly sclerotized, usually without styli; bursa copulatrix with infundibulum or fleshy lobe, with sperm duct composed of two parts of different diameter; spermatheca bean-shaped, sclerotised without well differentiated nodulus or ramus, with large accessory gland (after Ślipiński 2007).

##### Key to the Iranian species and genera of Chilocorini

**Table:** 

1	Fronto-clypeal plate emarginate anteriorly (Fig. 14). Postcoxal line on abdominal ventrite 1 merging with posterior margin of ventrite or running very close to it (Fig. 15). All tibiae with tooth at outer side; tibial spurs absent (Fig. 16). Elytron brown or reddish brown with 3 small orange discal spots in transverse row, usually partially fused (Fig. 2). Male genitalia with penis guide as long as parameres (Figs 17, 18), penis as in Figs 19, 20. (Body circular, strongly convex, 3.5–4.5 mm long)	***Chilocorus bipustulatus* Linnaeus**
–	Fronto-clypeal plate not emarginate. Postcoxal line on abdominal ventrite 1 distant from posterior margin of ventrite (Figs 21, 22). Mid-and hind tibiae smoothly arcuate; with 2 apical spurs (Fig. 23)	**2**
2	Antenna composed of 8 antennomeres (Fig. 24). Body yellow with two small black spots on each elytron, one behind the other (Fig. 1). Male genitalia with parameres slightly longer than penis guide (Fig. 25); penis as in Fig. 26. (Body broadly oval, 2.0–2.5 mm long)	***Brumoides adenensis* Fürsch**
–	Antenna composed of 10 antennomeres (Figs 27, 28)	**3**
3	Elytra black with red spots or red-brown with or without black spots. Body size 2.8–5.0 mm	***Exochomus* Redtenbacher 4**
–	Elytra completely black. Body size 2.2–4.5 mm	***Parexochomus* Barovsky 10**
4	Elytra black; each elytron with two small or medium sized, separated red spots	**5**
–	Elytra orange to red-brown, with or without black spots, or elytra black with large pale maculae of irregular shape	**6**
5	Each elytron with two similar and equally-sized rounded spots (Fig. 6). Male genitalia with penis guide approximately as long as parameres (Figs 29, 31); penis as in Fig. 30. Body oval, 3.5–4.5 mm long	***E. quadriguttatus* Fleischer**
–	Each elytron with two differently sized and shaped spots (Figs 7, 8). Male genitalia with penis guide clearly shorter than parameres (Figs 32, 33); penis as in Fig. 34. Body subcircular, 3.5–4.0 mm long	***E. quadripustulatus* Linnaeus**
6	Background of elytra black; elytral maculae large and of irregular shape, brown or orange	**7**
–	Background of elytra orange to red-brown; with or without contrasting markings	**8**
7	Humeral part with brown macula (Fig. 9); male genitalia with penis guide longer than parameres (Fig. 35); penis as in Fig. 36. Form oblong, body length 4.3–5.0 mm	***E. undulatus* Weise**
–	Humeral part with orange macula surrounding a black round spot (Fig. 3). Body form oblong, 3.0–5.0 mm long)	***E. ericae* Crotch**
8	Elytra brown without markings; (Body subcircular, 3.5–4.0 mm long)	***E. quadripustulatus* Linnaeus**
–	Each elytron with 4 nearly equally sized, small, black spots similarly distributed	**9**
9	Pronotum reddish orange, with a medio basal ungulate black spot (Fig. 5). Tarsal claw simple (Fig. 37). Male genitalia with penis guide as long as parameres (Fig. 38); penis as in Fig. 39. Body nearly of spindle form, 2.8–4.5 mm long	***E. octosignatus* Gebler**
–	Pronotum entirely black except for dark bordering oflateral and anterior margins (Fig. 4).Tarsal claw with small basal tooth (Fig. 40). Male genitalia with penis guide distinctly shorter than parameres (Figs 41, 42); penis as in Fig. 43. Body subcircular, 4.0–5.0 mm long	***E. gebleri* Weise**
10	Body pubescent	**11**
–	Body glabrous	**12**
11	Body covered with dense, moderately long setae (Fig. 13). Male genitalia with penis guide shorter than parameres (Figs 44, 45); penis as in Figs 46, 47. Body short oval to nearly circular, 2.8–2.9 mm long	***P. pubescens* Küster**
–	Body apparently glabrous, but actually with minute sparse setae particularly at pronotum (Fig. 10). Form oblong, 2.2–2.7 mm long	***P. melanocephalus* Zubkov**
12	Pronotum yellow (Fig. 11). Male genitalia as in Figs 48, 49, 50. Body oval and highly convex, 3.8–4.2 mm long	***P. nigripennis* Erichson**
–	Pronotum black with yellow lateral margins (Fig. 12). Male genitalia as in Figs 51–55. Body broadly oval, moderately convex, 3.1–4.5 mm long	***P. nigromaculatus* Goeze**

### Updated checklist of the Iranian species of Chilocorini

#### Brumoides

Taxon classificationAnimaliaColeopteraCoccinellidae

Chapin, 1965

Brumoides Chapin, 1965: 237. Type species: Coccinella suturalis Fabricius, 1798, by original designation.

##### Diagnosis.

Body length 2.0–3.5 mm. Dorsum glabrous; yellowish or brown, elytra with dark markings. Eye distinctly emarginate. Antenna composed of 8 antennomeres; terminal antennomere small, partly embedded in penultimate one. Clypeus short; labrum exposed. Pronotal base bordered; prosternal process extremely narrow, without carinae; without hypomeral fovea. Fore tibia narrow, simple, middle and hind tibiae with two apical spurs; tarsal claws appendiculate or weakly thickened basally. Abdominal ventrite 6 visible in males; abdominal postcoxal lines separated medially, each arcuately recurving apically and reaching or nearly reaching midpoint of lateral line (after Ślipiński 2007).

##### Ecology.

Various species of *Brumoides* have been associated with mealybugs (Ślipiński 2007), namely *Coccidohystrix insolita* (Hemiptera: ), *Dactylopius confusus* (Hemiptera: Dactylopiidae), *Ferrisia virgata* (Hemiptera: Pseudococcidae), and *Phenacoccus solenopsis* (Hemiptera: ) (Gordon 1985, Gautam 1990, Hodek and Honěk 2009, Arif et al. 2012, Giorgi et al. 2014). Some species of this genus, such as *Brumoides suturalis* (F.) feed on some whitefly species, such as *Aleurolobus barodensis* (Maskell) (Inayatullah 1984, Hodek and Honěk 2009) in addition to feeding on some coccids, such as *F. virgata* (better for development) and *Planococcus pacificus* (better for oviposition) (Gautam 1990).

#### Brumoides adenensis

Taxon classificationAnimaliaColeopteraCoccinellidae

Fürsch, 1987

Figs 1 , 21 , 24–26

Brumoides adenensis Fürsch, 1987: 44.

##### General distribution.

Middle East (that includes Iran, Saudi Arabia, United Arab Emirates, Yemen) (Kovář 2007), Southern Africa (Łączyński and Tomaszewska 2012).

##### Distribution in Iran.

Iran (Kovář 2007) – no specific distribution known.

##### Remarks.

The species descriptions and photographs by Fürsch (1987) and Raimundo et al. (2008) were used with some modifications.

#### Chilocorus

Taxon classificationAnimaliaColeopteraCoccinellidae

Leach, 1815

Chilocorus Leach, 1815: 116. Type species: Coccinella cacti Linnaeus, 1767, by monotypy.

##### Diagnosis.

Body length 2.5–4.8 mm. Dorsal body glabrous; elytra black or brown with white or orange markings; eye clearly emarginate. Antennae short, composed of 8 antennomeres; with scape symmetrical; 8^th^ antennomere either as long as or markedly longer than antennomere 7. Clypeus long; labrum partly exposed. Pronotal base unbordered; prosternal process narrow without carinae; hypomeral fovea absent. All tibiae flattened and angulate externally, without apical spurs; tarsal claws strongly appendiculate. Elytral margin not reflexed with indistinct bead; epipleural foveae weak. Abdominal ventrite 6 visible in males; abdominal postcoxal lines separated medially, each running parallel to hind margin of ventrite (after Ślipiński 2007).

##### Ecology.

Although various scale insects are primary hosts of *Chilocorus* (Escalona et al. 2017), some species at least accept aphids as prey (Gordon 1985, Drea and Gordon 1990, Ślipiński 2007, Hodek and Honěk 2009). Nonetheless, there are some reports about some species of this genus, such as *Chilocorus stigma* (Say) which feed on some whitefly species, such as *Aleurocanthus woglumi* Ashby (Dowell and Cherry 1981, Hodek and Honěk 2009).

**Figures 1–9.**
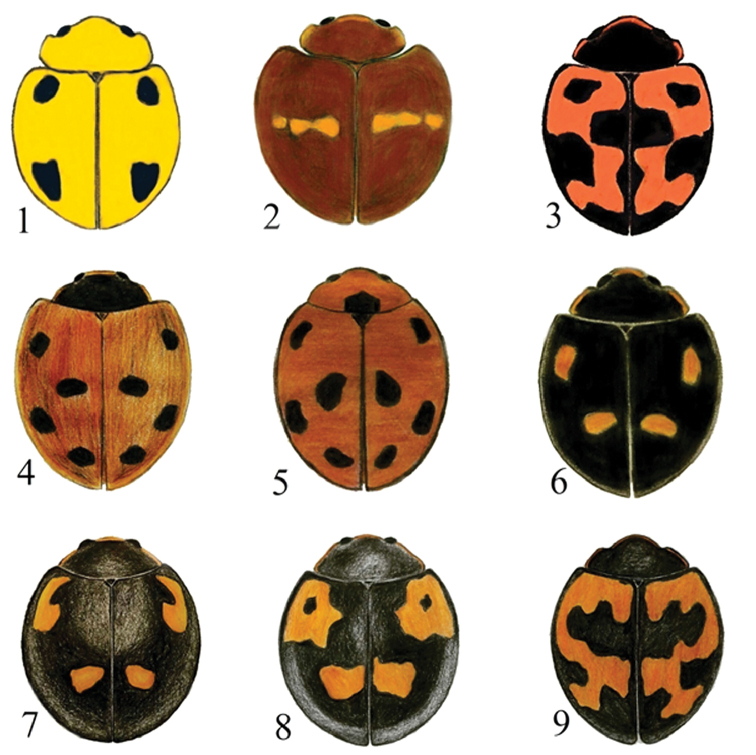
Dorsal habitus of Chilocorini species. **1** *Brumoides adenensis* Fürsch **2** *Chilocorus bipustulatus* Linnaeus **3** *E. ericae* Crotch **4** *E. gebleri* Weise **5** *E. octosignatus* Gebler **6** *E. quadriguttatus* Fleischer **7, 8** *E. quadripustulatus* Linnaeus **9** *E. undulatus* Weise.

#### Chilocorus bipustulatus

Taxon classificationAnimaliaColeopteraCoccinellidae

(Linnaeus, 1758)

Figs 2 , 16 , 15–20

Coccinella bipustulata Linnaeus, 1758: 367.
Coccinella fasciata Müller, 1776: 68.
Coccinella transversoguttata Börner, 1776: 250.
Coccinella frontalis Thunberg, 1792: 105. [Homonym]
Coccinella testudo Florencourt Chassot, 1796: 214.
Coccinella strigata Fabricius, 1798: 79. [Homonym]
Chilocorus olivetorum Costa, 1839: 104.
Chilocorus minor Sahlberg, 1903: 86.

##### Material examined.

8♂, 3♀: Iran, Lorestan province, V.2013, lgt. Amir Biranvand, det. Biranvand. 2♂, 1♀: Iran, Semnan province, V.2015, lgt. Mino Toozandejani, det. Biranvand.

##### General distribution.

Afrotropical region, Nearctic region, Palaearctic region (Mader 1955, Gordon 1985, Kovář 2007, Canepari 2011) and Oriental region (Poorani 2002).

##### Distribution in Iran.

Widely distributed (Duverger 1983, Moddarres-Awal 2012).

##### Ecology.

This species feeds on a wide range of Hemiptera species: *Agonoscena pistaciae* (Psyllidae), *Aonidiella orientalis* (Diaspididae), *Bemisia tabaci* (Aleyrodidae), *Chrysomphalus dictyospermi* (Diaspididae), *Eulecanium prunastri* (Coccidae), *Euphyllura olivina* (Psyllidae), *Salicola kermanensis* (Diaspididae), *Lepidosaphes malicola* (Diaspididae), *Leucaspis pusilla* (Diaspididae), *Maconellicoccus hirsutus* (Pseudococcidae), *Ommatissus binotatus lybicus* (Tropiduchidae), *Parlatoria blanchardi* (Diaspididae), *Parlatoria oleae* (Diaspididae), *Phloeomyzus passerinii* (Aphididae), *Planococcus citri* (Pseudococcidae), *Pseudaulacaspis pentagona* (Diaspididae), *Psylla pyricola* (Psyllidae) (Moddarres-Awal 2012) and other coccids, particularly armoured scales (Hodek 1973, Stansly 1984).

**Figures 10–13.**
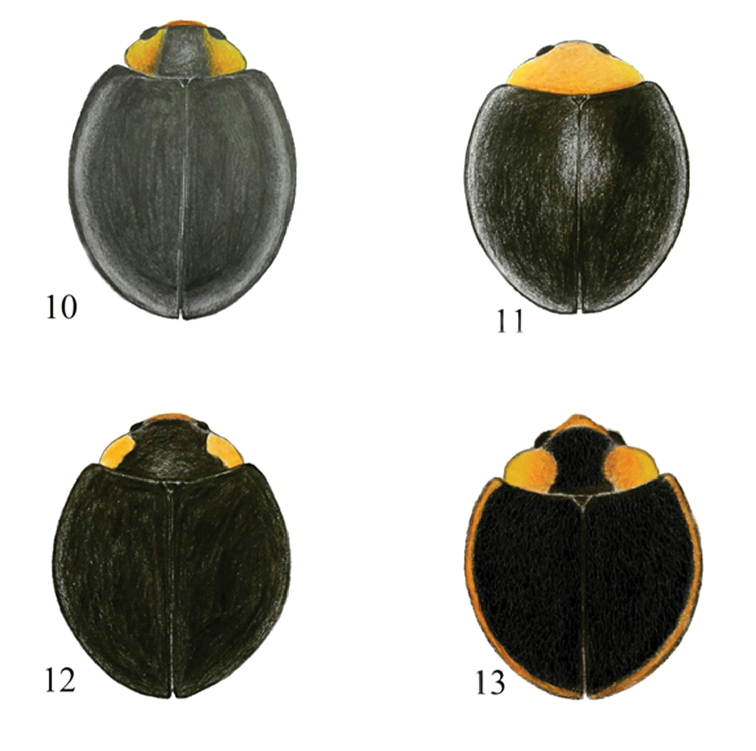
Dorsal habitus of Chilocorini species. **10** *Parexochomus melanocephalus* Zubkov **11** *P. nigripennis* Erichson **12** *P. nigromaculatus* Goeze **13** *P. pubescens* Küster.

#### Exochomus

Taxon classificationAnimaliaColeopteraCoccinellidae

Redtenbacher, 1843

Exochomus Redtenbacher, 1843:11. Type species: Coccinella quadripustulata Linnaeus, 1758, by subsequent designation of Thomson, 1859.

##### Diagnosis.

Body length 2.8–5.5 mm. Dorsal body glabrous; elytra black, brown, or yellow, often with contrasting red or yellow markings; sometimes (in lighter coloured species) with black stripes along lateral margins of elytra. Antenna composed of 10 antennomeres, minute terminal antennomere embedded in penultimate one; pronotal basal margin completely bordered with submarginal line; prosternal process narrow, truncate apically, without carinae; elytral epipleura clearly narrowing, without foveae; abdominal postcoxal lines complete or nearly so, semicircular, reaching to inner end of lateral line; meso- and metatibiae each with two apical spurs; tarsal claws with or without basal tooth (after Li et al. 2015).

##### Ecology.

Most species of this genus are aphidophagous and coccidophagous (Gordon 1985, Kovář 1995, Magro et al. 2010). Nontheless, there are some reports about some species of the genus feeding on aleyrodids e.g., *Exochomus bimaculosus* Mulsant which feeds on *Bemisia tabaci* (Gennadius) (Yigit 1992, Leite et al. 2003, Hodek and Honěk 2009).

**Figures 14–20.**
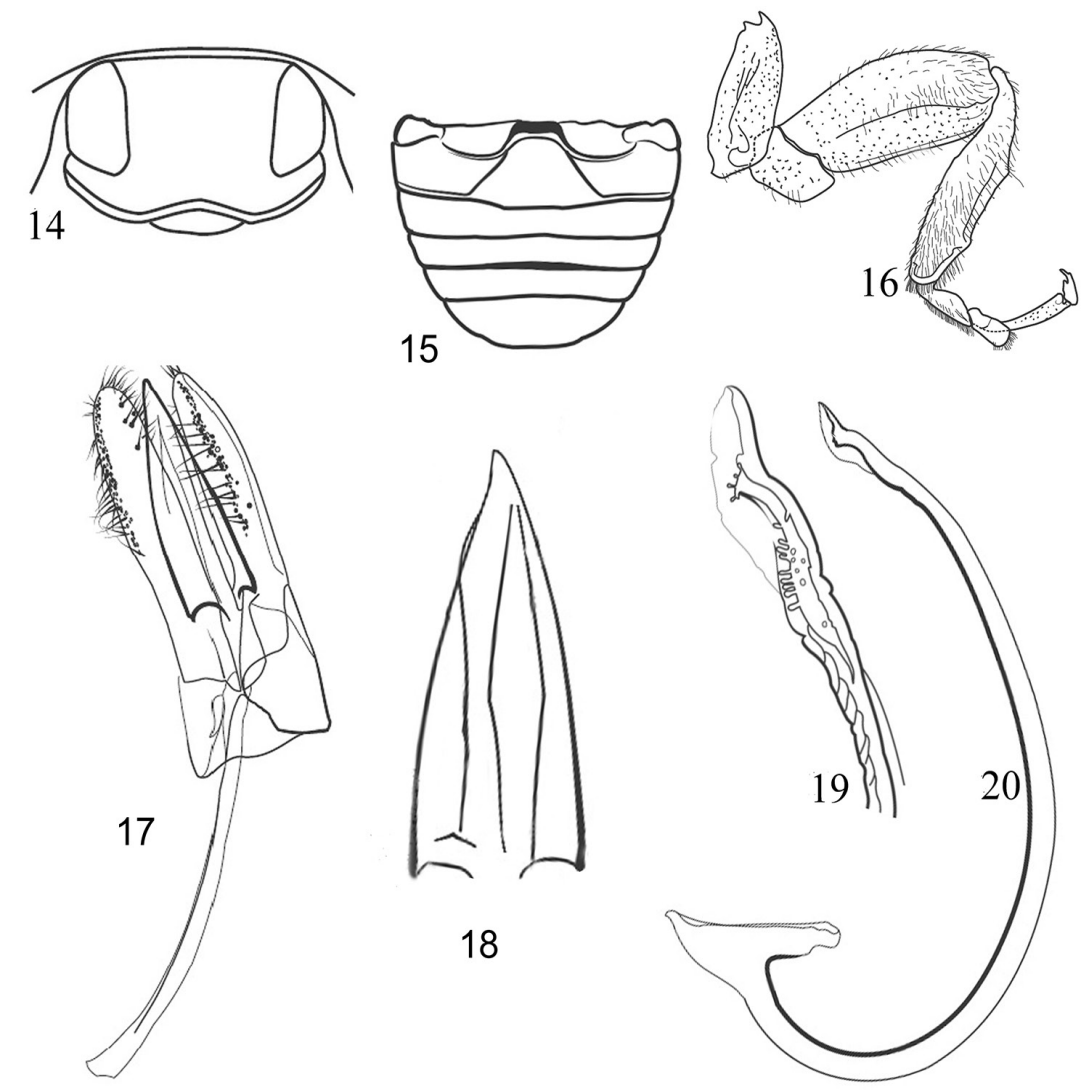
Morphological details and male genitalia of Chilocorini species. **14–20** *Chilocorus bipustulatus*: **14** Head **15** Abdominal postcoxal lines **16** Leg **17** Tegmen **18** Penis guide of tegmen **19** Penis apex **20** Penis.

#### Exochomus bifasciatus

Taxon classificationAnimaliaColeopteraCoccinellidae

Barovsky, 1927

Exochomus bifasciatus Barovsky, 1927: 200.

##### General distribution.

China, Iran, Kazakhstan (Kovář 2007).

##### Distribution in Iran.

Iran (Kovář 2007) – no specific distribution provided.

#### Exochomus ericae

Taxon classificationAnimaliaColeopteraCoccinellidae

Crotch, 1874

Fig. 3

Exochomus ericae Crotch, 1874: 193.
Chilocorus nigropictus Fairmaire, 1876: 94.
Chilocorus picturatus Fairmaire, 1876: 94.
Exochomus anchorifer Allard, 1870: 9.

##### General distribution.

Algeria, Iran, Morocco, Tunisia (Mader 1955, Duverger 1983, Kovář 2007).

##### Distribution in Iran.

Dasht Arzhanregion, Kerman, Nowshahr region (Duverger 1983).

##### Remarks.

We used the species descriptions and photographs of Mader (1955) with some modifications.

**Figures 21–31.**
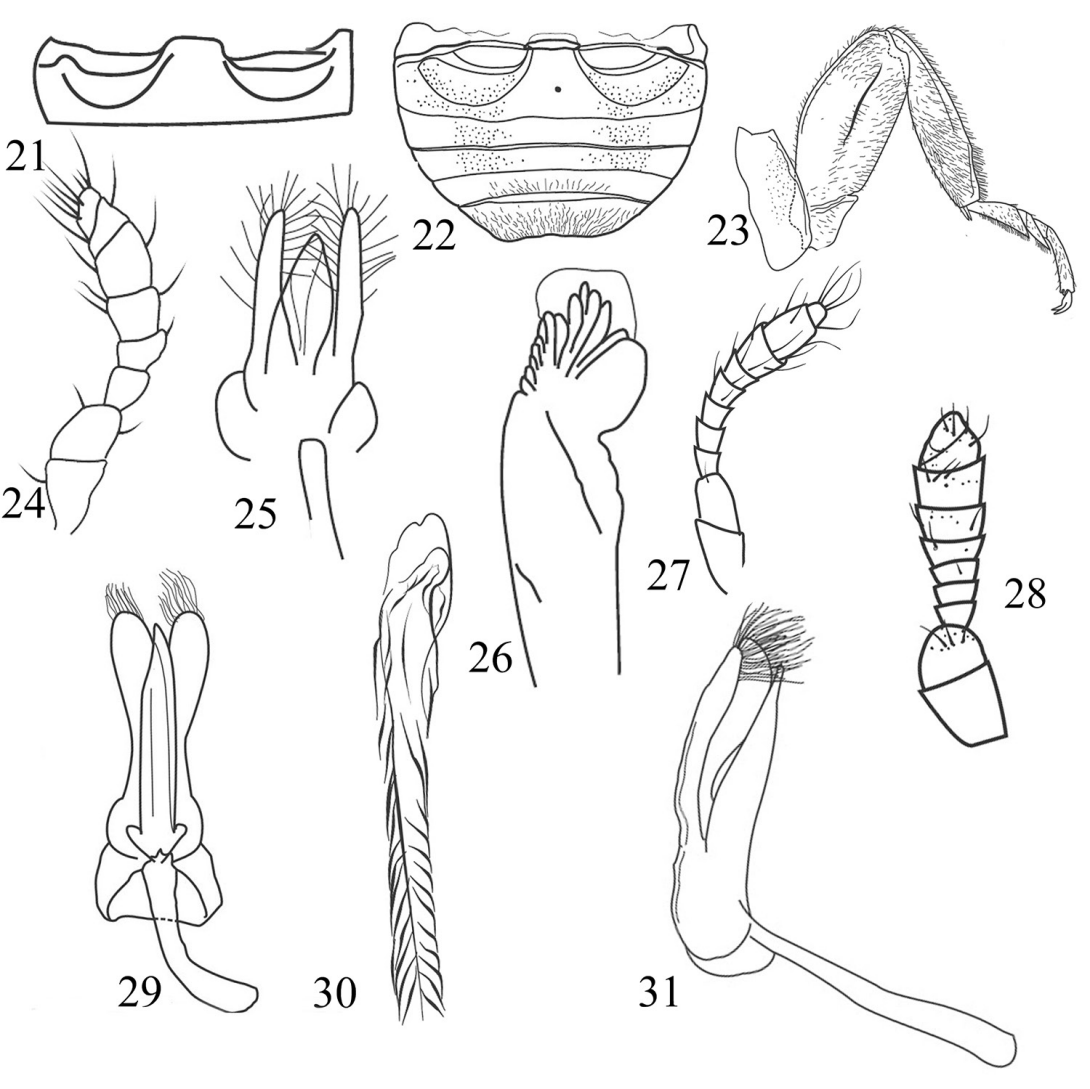
Morphological details and male genitalia of Chilocorini species. **21**, **24–26** *Brumoides adenensis*: **21** Abdominal postcoxal lines **24** Antenna **25** Tegmen **26** Penis apex **22** *Parexochomus pubescens*: Abdominal postcoxal lines **23, 28** *P. nigripennis*: **23** Hind leg **28** Antenna **27** *Exochomus undulatus*: Antenna **29–31** *E. quadriguttatus*: **29** Tegmen, ventral view **31** Tegmen, lateral view **30** Penis apex.

#### Exochomus gebleri

Taxon classificationAnimaliaColeopteraCoccinellidae

Weise, 1885

Figs 4 , 40–43

Exochomus gebleri Weise, 1885: 55.

##### Material examined.

5♂, 2♀: Iran, Yazd province, spring and summer 2013, lgt. Mehdi Zare Khormizi, det. Biranvand.

##### General distribution.

Afghanistan, Iran, Turkey (Kovář 2007).

##### Distribution in Iran.

Golestan, Semnan (Kovář 1995), Lorestan (Jafari and Kamali 2007), Fars (Moddarres-Awal 2012), Yazd (current study).

#### Exochomus octosignatus

Taxon classificationAnimaliaColeopteraCoccinellidae

(Gebler, 1830)

Figs 5 , 37–39

Coccinella octosignata Gebler, 1830: 225.
Coccinella deserta Motschulsky, 1840: 175.
Coccinella desertorum Gebler, 1841: 376.
Brumus lasioides Weise, 1879: 135.
Brumus conjunctus Fleischer, 1900: 118.

##### General distribution.

Afghanistan, Armenia, Azerbaijan, France, Iran, Iraq, Italy, Kazakhstan, Kyrgyzstan, Mongolia, Russia, Tajikistan, Turkmenistan, Turkey, Uzbekistan (Kovář 2007).

##### Distribution in Iran.

Khameshorkn region (Duverger 1983), Khorasan (Moodi and Mossadegh 1995, Yaghmaei and Kharrazi Pakdel 1995), Chaharmahal and Bakhtiari (Bagheri and Mossadegh 1996), East Azerbaijan, Gilan, Isfahan, Kerman, Qom, Tehran, Sistan and Baluchestan (Moddarres-Awal 2012).

##### Ecology.

This species feeds on the mealybugs *Phenacoccus aceris* and *Planococcus citri* (Pseudococcidae) (Moddarres-Awal 2012).

**Figures 32–39.**
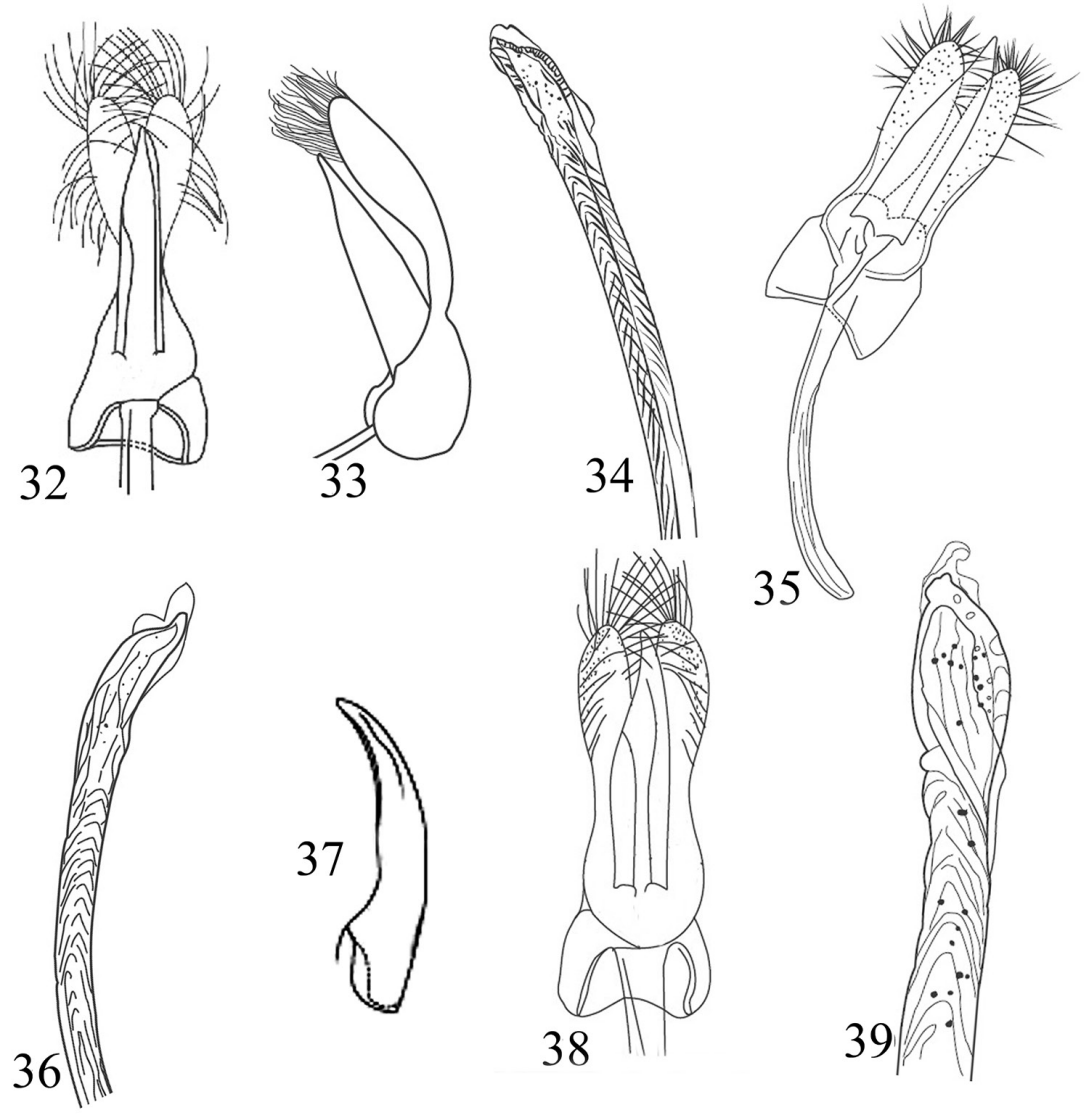
Morphological details and male genitalia of Chilocorini species **32–34** *E. quadripustulatus*: **32, 33** Tegmen in ventral and lateral view **34** Penis apex **35–36** *E. undulatus*: **35** Tegmen, ventral view **36** Penis apex **37–39** *E. octosignatus*: **37** Tarsal claw **38** Tegmen, ventral view **39** Penis apex.

#### Exochomus quadriguttatus

Taxon classificationAnimaliaColeopteraCoccinellidae

Fleischer, 1900

Figs 6 , 29–31

Exochomus quadriguttatus Fleischer, 1900: 118.
Exochomus cordiformis Roubal, 1926: 245.
Exochomus illaesicollis Roubal, 1927: 135.

##### Material examined.

3♂, 8♀: Iran, Semnan province, VII.2015, lgt. Mino Toozandejani, det. Biranvand.

##### General distribution.

Caucasus, Iran, Lebanon, Syria (Duverger 1983), Armenia, Turkey (Kovář 2007).

##### Distribution in Iran.

Sagdar region (Duverger 1983), Kerman (Moddarres-Awal 2012), Semnan (current study).

#### Exochomus quadripustulatus

Taxon classificationAnimaliaColeopteraCoccinellidae

(Linnaeus, 1758)

Figs 7–8 , 32–34

Coccinella quadripustulata Linnaeus, 1758: 367.
Coccinella lunulata Gmelin, 1790: 1662.
Coccinella quadriverrucata Fabricius, 1792: 288.
Coccinella cassidoides Donovan, 1798: 74.
Coccinella varia Schrank, 1798: 444.
Coccinella distincta Brullé, 1832: 273
Coccinella iberica Motschulsky, 1837: 422.
Coccinella floralis Motschulsky, 1837: 423.
Exochomus haematideus Costa, 1849: 62.
Exochomus unicolor Schaufuss, 1862: 50
Exochomus sexpustulatus Kraatz, 1873:192
Exochomus bilunulatus Weise, 1879: 133.
Exochomus koltzei Weise, 1879: 134.
Exochomus reitteri Schneider, 1881: 16
Exochomus vittatus Fuente, 1910: 444

##### Material examined.

60♂, 75♀: Iran, Lorestan province, in all seasons, 2013, 2014, 2015, 2016, 2017, lgt. Amir Biranvand, det. Biranvand. 3♂, 3♀: Iran, Semnan province, V.2015, lgt. Mino Toozandejani, det. Biranvand.

##### General distribution.

Palaearctic Region, Oriental region, Australian region, Nearctic region (USA: California) (Canepari 2011, Li et al. 2015).

##### Distribution in Iran.

Widely distributed (Duverger 1983, Moddarres-Awal 2012).

##### Ecology.

This species feeds on various species of Hemiptera, namely: *Aonidiella orientalis* (Diaspididae), *Aphis fabae* (Aphididae), *Callaphis juglandis* (Aphididae), *Chromaphis juglandicola* (Aphididae), *Eriosoma lanigerum* (Aphididae), *Eulecanium prunastri* (Coccidae), *Euphyllura olivina* (Psyllidae), *Maconellicoccus hirsutus* (Pseudococcidae), *Parlatoria oleae* (Diaspididae), *Psylla pyricola* (Psyllidae), *Saissetia oleae* (Coccidae) (Moddarres-Awal 2012), and other aphids and Coccidae (Uygun 1981, Ülgentürk and Toros 2001, Kaydan et al. 2006, Kaydan et al. 2012).

**Figures 40–47.**
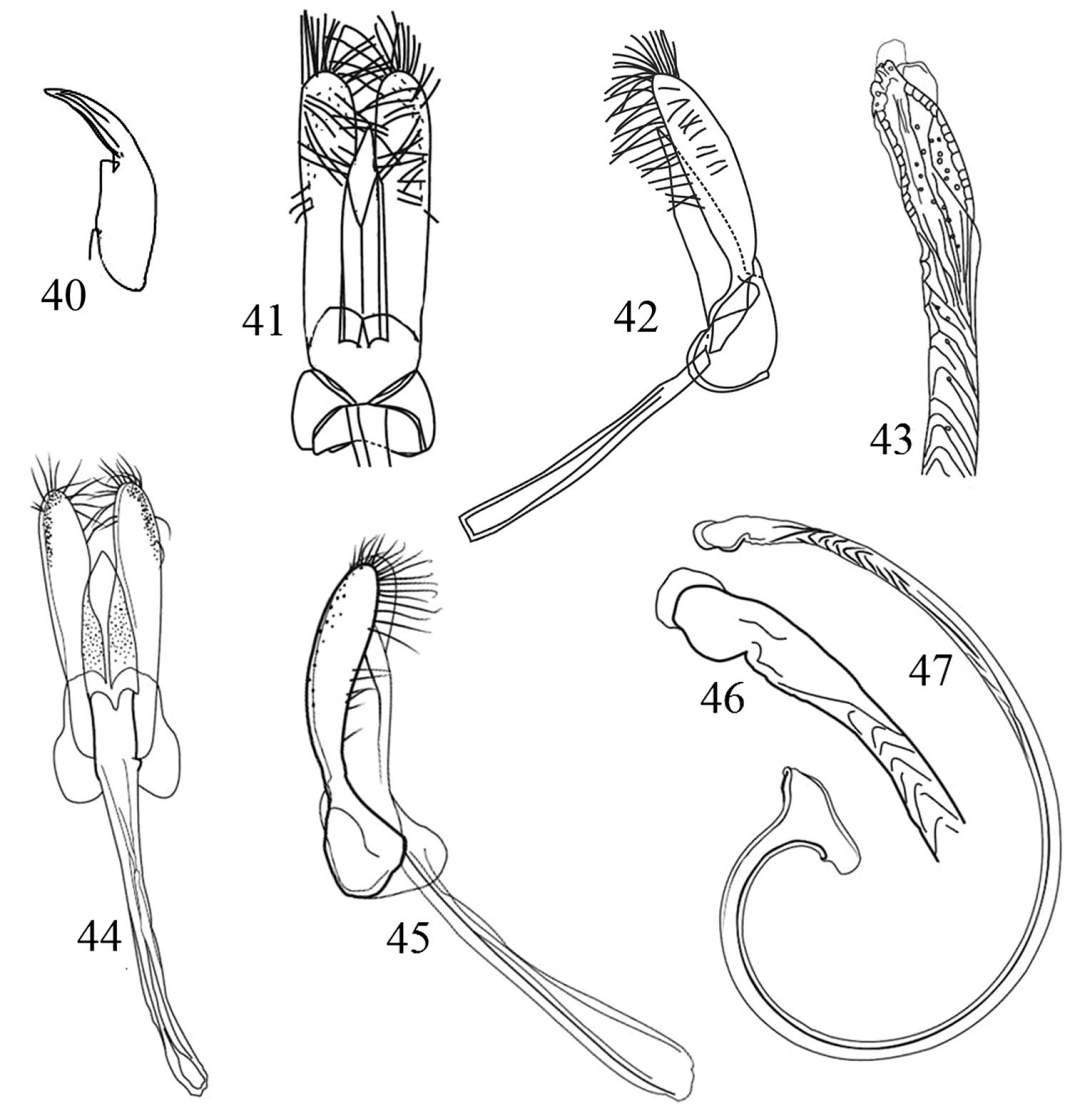
Morphological details and male genitalia of Chilocorini species. **40–43** *E. gebleri*: **40** Tarsal claw **41, 42** Tegmen in ventral and lateral view **43** Penis apex **44–47** *P. pubescens*: **44–45** Tegmen in ventral and lateral view **46** Tip of penis **47** Penis.

#### Exochomus undulatus

Taxon classificationAnimaliaColeopteraCoccinellidae

Weise, 1878

Figs 9 , 27 , 35–36

Exochomus undulatus Weise, 1878: 93

##### Material examined.

10♂, 16♀: Iran, Lorestan province, in all seasons, 2013, 2015, 2016, lgt. Amir Biranvand, det. Biranvand.

##### General distribution.

Palestine (Mader 1955), Caucasus (Duverger 1983), Afghanistan, Azerbaijan, Egypt, Georgia, Iraq, Iran, Lebanon, Syria, Tajikistan (Kovář 2007).

##### Distribution in Iran.

Lorestan (Jafari and Kamali 2007), Chaharmahal and Bakhtiari, Fars, Isfahan, Kerman, Khorasan, Kohgiluyeh and Boyer-Ahmad, Qazvin (Moddarres-Awal 2012), Tehran (Ghanbari et al. 2012), Markazi (Ahmadi et al. 2012), Yazd (Zare Khormizi et al. 2016).

##### Ecology.

This species feeds usually on *Euphyllura olivina* (Hemiptera: Psyllidae) (Moddarres-Awal 2012).

#### Parexochomus

Taxon classificationAnimaliaColeopteraCoccinellidae

Barovsky, 1922

Exochomus (Parexochomus) Barovsky, 1922: 293. Type species: Exochomus pubescens Küster, 1848, by subsequent designation of Chapin 1965.
Parexochomus : Kovář 2007: 595.

##### Diagnosis.

Body length 3.0–3.5 mm. Dorsal body glabrous or pubescent, dark brown or black with lateral margins of pronotum or at least anterior angles yellow or red. Antenna composed of 10 antennomeres, minute terminal antennomere embedded in penultimate one; terminal maxillary palpomeres stout, nearly parallel-sided; pronotal basal margin entirely bordered with submarginal line; prosternal process narrow, rounded apically, without carinae; elytral epipleura clearly narrowing towards apex, without foveae; abdominal postcoxal lines complete and semicircular, reaching to middle of lateral line; meso- and metatibiae each with two apical spurs; tarsal claws with basal tooth (after Li et al. 2015).

##### Ecology.

The species of *Parexochomus* are aphidophagous or coccidophagous (Moddarres-Awal 2012).

#### Parexochomus melanocephalus

Taxon classificationAnimaliaColeopteraCoccinellidae

(Zubkov, 1833)

Fig. 10

Coccinella melanocephala Zubkov, 1833: 339.
Exochomus russicollis Mulsant, 1850: 1033.

##### General distribution.

Southern Russia, Caucasus (Mader 1955), Azerbaijan, Armenia, Bulgaria, Georgia, Iran, Kazakhstan, Tajikistan, Turkmenistan, Turkey, Uzbekistan (Kovář 2007).

##### Distribution in Iran.

Razavi Khorasan (Yaghmaei and Kharrazi Pakdel 1995), Lorestan (Jafari and Kamali 2007), Chaharmahal and Bakhtiari, Khorasan (Moddarres-Awal 2012), Kerman (Salehi et al. 2011), Hormozgan (Fallahzadeh et al. 2013).

#### Parexochomus nigripennis

Taxon classificationAnimaliaColeopteraCoccinellidae

(Erichson, 1843)

**Figures 48–55.**
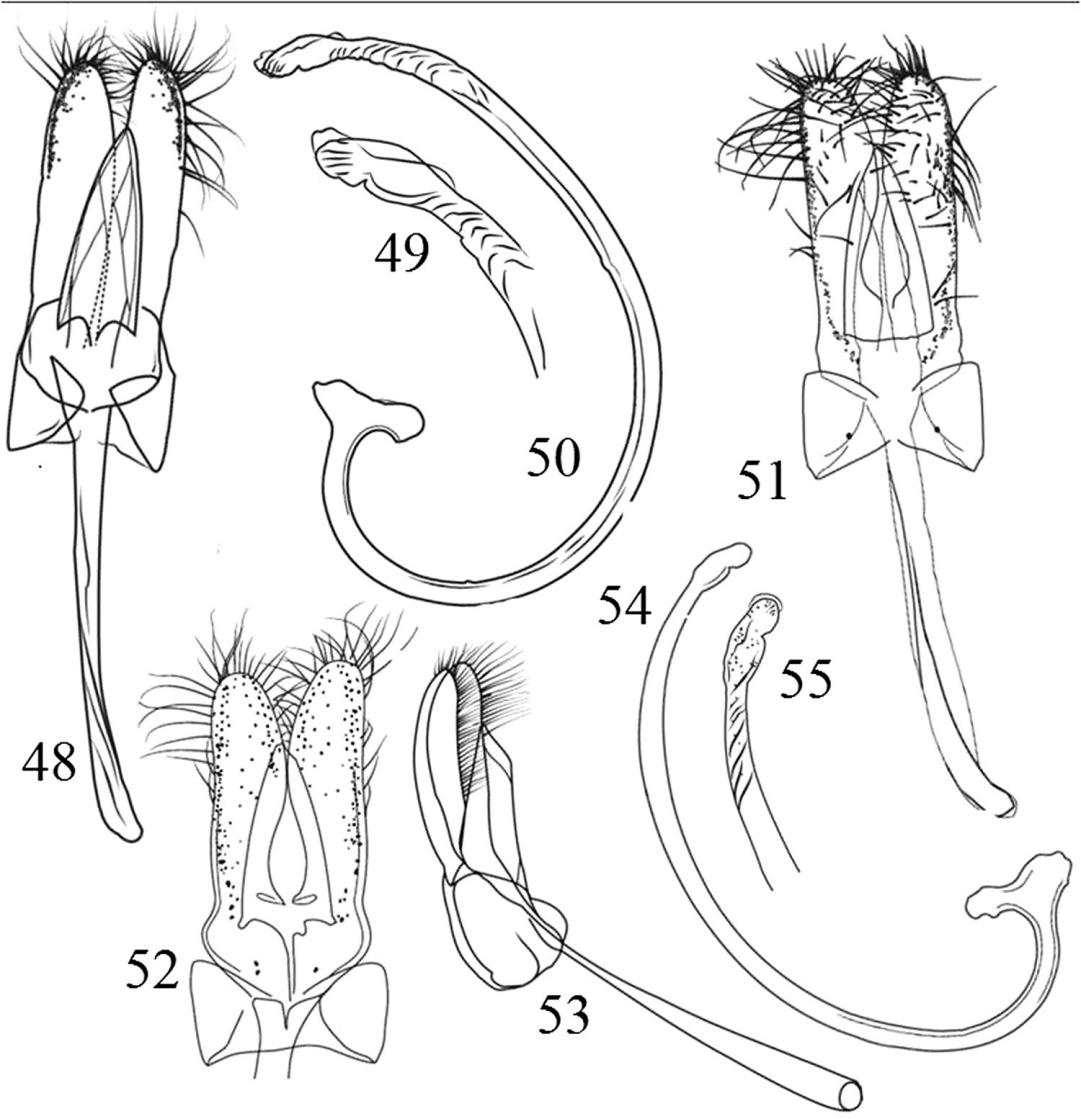
Morphological details and male genitalia of Chilocorini species. **48–50** *P. nigripennis*: **48** Tegmen, ventral view **49** Penis apex **50** Penis **51–55** *P. nigromaculatus*: **51–53** Tegmen, ventral and lateral view **54** Penis **55** Penis apex.

Figs 11 , 23 , 28 , 48–50

Chilocorus nigripennis Erichson, 1843: 267.
Exochomus xanthoderus Fairmaire, 1864: 648.

##### Material examined.

10♂, 16♀: Iran, Lorestan province, VII.2014, lgt. Amir Biranvand, det. Biranvand.

##### General distribution.

Oriental region (Poorani 2002), Afrotropical region, Mediterranean region, Middle East (Kovář 2007).

##### Distribution in Iran.

Golestan (Montazeri and Mossadegh1995), Lorestan (Jafari and Kamali 2007), Gilan (Hajizadeh et al. 2003), Fars, Kerman, Khorasan, Khuzestan, Sistan, and Baluchestan (Moddarres-Awal 2012), Lorestan (current study).

##### Ecology.

This species feeds usually on the following hemipterans: *Acanthococcus abaii* (Eriococcidae), *Agonoscena pistaciae* (Psyllidae), *Bemisia tabaci* (Aleyrodidae) (Moddarres-Awal 2012).

#### Parexochomus nigromaculatus

Taxon classificationAnimaliaColeopteraCoccinellidae

(Goeze, 1777)

Figs 12 , 51–55

Coccinella nigromaculata Goeze, 1777: 248. Coccinella testudinare Geoffroy in Fourcroy, 1785: 151. Coccinella aurita Scriba, 1791: 101. Coccinella humerale Townson, 1800: 167.
Chilocorus rufipes Stephens, 1832: 375. Exochomus collaris Küster, 1849: 100. Exochomus pyrenaeus Kraatz, 1873: 194.

##### Material examined.

75♂, 90♀: Iran, Lorestan province, spring and summer 2013, 2014, 2015, 2016, 2017, lgt. Amir Biranvand, det. Biranvand. 3♂, 1♀: Iran, Semnan province, VI.2015, lgt. Mino Toozandejani, det. Biranvand.

##### General distribution.

Palaearctic region (Duverger 1983, Kovář 2007).

##### Distribution in Iran.

Widely distributed (Duverger 1983, Moddarres-Awal 2012).

##### Ecology.

This species feeds usually on the following species of Hemiptera: *Agonoscena pistaciae* (Psyllidae), *Aonidiella orientalis* (Diaspididae), *Bemisia tabaci* (Aleyrodidae), *Diuraphis noxia* (Aphididae), *Eulecanium prunastri* (Coccidae), *Euphyllura olivina* (Psyllidae), *Maconellicoccus hirsutus* (Pseudococcidae), *Therioaphis maculata* (Aphididae) (Moddarres-Awal 2012) and other aphids and Coccidae (Uygun 1981, Atlıhan and Özgökçe 2002, Kaydan et al. 2012).

#### Parexochomus pubescens

Taxon classificationAnimaliaColeopteraCoccinellidae

(Küster, 1848)

Figs 13 , 22 , 44–47

Exochomus pubescens Küster, 1848: 94
Exochomus apicatus Fairmaire, 1884: 59.
Exochomus circumcinctus Sahlberg, 1903: 36.
Platynaspis flavilabris Motschulsky, 1849: 155.
Platynaspis flavilabris Mulsant, 1850b: 947. [Homonym]
Exochomus gestroi Fairmaire, 1875: 540.
Exochomus lugubrivestis Mulsant, 1853: 194.
Exochomus saharae Sicard, 1929: 60

##### Material examined.

3♂, 5♀: Iran, Lorestan province, VII.2014, lgt. Amir Biranvand, det. Biranvand.

##### General distribution.

Oriental region, Palestine, Syria (Poorani 2002), Afghanistan, Algeria, Egypt, France, Greece, Iran, Israel, Italy, Libya, Morocco, Saudi Arabia, Spain, Tunisia (Kovář 2007).

##### Distribution in Iran.

Angohran region, Hormozgan, Tehran (Karaj), Khuzestan (Susangerd), Ramine region, Daran region, Sagdan region (Duverger 1983), Lorestan (Jafari and Kamali 2007), Fars, Kerman, Khorasan, Khuzestan, Sistan, and Baluchestan (Moddarres-Awal 2012).

##### Ecology.

This species feeds on *Bemisia tabaci* (Hemiptera: Aleyrodidae) and *Tetranychus turkestani* (Acari) (Moddarres-Awal 2012).

## Supplementary Material

XML Treatment for Chilocorini

XML Treatment for Brumoides

XML Treatment for Brumoides adenensis

XML Treatment for Chilocorus

XML Treatment for Chilocorus bipustulatus

XML Treatment for Exochomus

XML Treatment for Exochomus bifasciatus

XML Treatment for Exochomus ericae

XML Treatment for Exochomus gebleri

XML Treatment for Exochomus octosignatus

XML Treatment for Exochomus quadriguttatus

XML Treatment for Exochomus quadripustulatus

XML Treatment for Exochomus undulatus

XML Treatment for Parexochomus

XML Treatment for Parexochomus melanocephalus

XML Treatment for Parexochomus nigripennis

XML Treatment for Parexochomus nigromaculatus

XML Treatment for Parexochomus pubescens
